# Huge Interatrial Septal Aneurysm: A Coincidental but Rather Fatal Finding

**DOI:** 10.1155/2011/705260

**Published:** 2011-01-12

**Authors:** Petros Tzimas, Georgios Papadopoulos

**Affiliations:** Department of Anesthesia and Postoperative Intensive Care, School of Medicine, University Hospital of Ioannina, University of Ioannina, 451 10 Ioannina, Greece

## Abstract

We report the case of a patient with a huge interatrial septal aneurysm as an intraoperative coincidental finding that led to a fatal outcome. The patient was admitted to our hospital in order to undergo elective coronary artery bypass grafting because he suffered from severe coronary artery disease. We intraoperatively diagnosed by transesophageal echocardiography a huge interatrial septal aneurysm mimicking a right atrial tumor. The aneurysm was initially resected and then coronary artery bypass grafting was successfully performed but the patient never achieved a successful separation from cardiopulmonary bypass probably because of massive embolic events.

## 1. Introduction

Atrial septal aneurysm (ASA) is no longer a rare clinical entity because of the improvement in two-dimensional echocardiography and the widespread use of transesophageal echocardiography (TEE). According to several studies using TEE, the prevalence of ASA ranges between 2% and 10% [1, 2]. Other types of cardiac abnormalities frequently associated with ASA are Atrial Septal Defects (ASDs) and Patent Foramen Ovale (PFO) as well as Mitral Valve Prolapse and atrial arrhythmias [3–5]. Many studies have established ASA as a risk factor for thromboembolic events, especially in association with PFO [6, 7]. In our case, we report a situation in which a patient with a huge undiagnosed ASA, associated with ASD and PFO, presented in the operating theatre in order to undergo CABG surgery. The patient had never experienced any thromboembolic events, despite his advanced age. However, this patient never achieved separation from CBP probably because of massive embolic events which occurred mainly during cannulation.

## 2. Case Presentation

**Figure 1:**
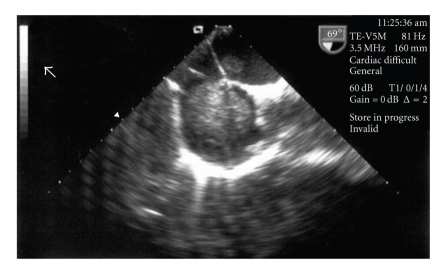
TEE image showing a very large echogenic mass in the right atrium.

**Figure 2:**
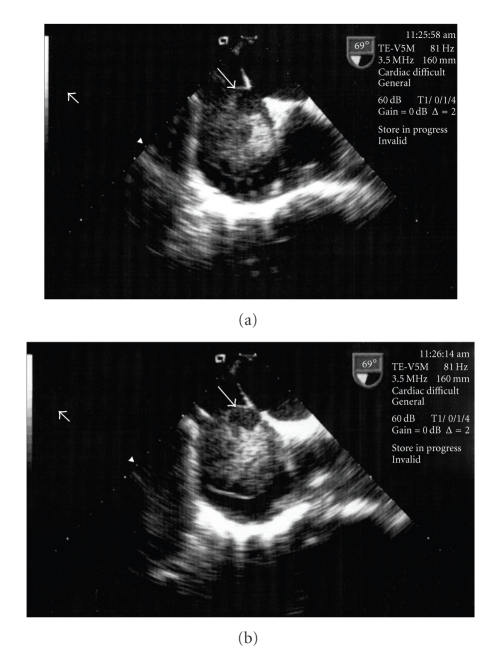
(a) TEE image demonstrating the detachment of part of the thrombus (arrow). (b) TEE image demonstrating the detachment of part of the thrombus (arrow).

**Figure 3:**
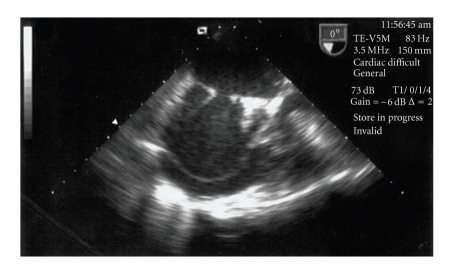
TEE recording where the thrombi are not visible.

**Figure 4:**
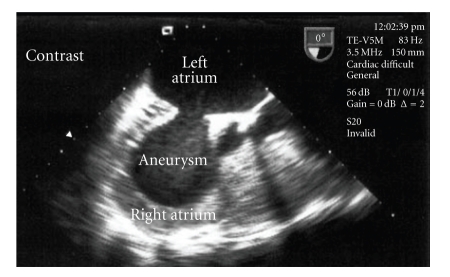
TEE revealing a huge atrial septal aneurysm with automated contrast after bolus injection of agitated saline in the right jugular vein.

A 75-year-old man was scheduled for elective coronary artery bypass grafting (CABG) due to coronary artery disease. His past medical history was notable for chronic atrial fibrillation and he had had a history of stable coronary artery disease for five years but he complained of angina at rest during the last two months before admission to our institution. His medications included aspirin, beta blockers, and nitrates. However, this patient was not receiving any anticoagulation. On admission to the hospital the patient underwent a transthoracic echocardiography that showed mild left ventricular systolic dysfunction with ejection fraction of 40% to 45% and mild dilation of both atria. The presence of interatrial septal aneurysm was not noticed. Coronary angiography revealed severe stenosis of the proximal left anterior descending artery. The EuroSCORE Logistic was estimated at 4.36%. A transesophageal echocardiography was performed after induction of anesthesia and tracheal intubation, and revealed a large echogenic mass in the right atrium (Figure 1). The mass was at first thought to be a large myxoma or probably thrombus attached to the atrial septum. Continuing with TEE examination, a visible deficiency was observed in the huge mass which was attached to the atrial septum. This was possibly due to a detachment of part of the thrombus (Figures 2(a) and 2(b)). The extraction of the echogenic parts towards the left atrium raised the suspicion of an atrial septal aneurysm which was completely filled with thrombi. In the following echo recordings, these thrombi were not visible any more (Figure 3). A huge atrial septal aneurysm was revealed, after bolus injection of agitated saline in the right jugular vein with automated contrast (Figure 4) and having used provocation manoeuvres including ventilation with PEEP (15 cm H_2_O) and high tidal volume, a patent foramen ovale was established. Afterwards, during cannulation for cardiopulmonary bypass the patient became haemodynamically unstable and was urgently introduced into extracorporeal circulation. After institution of cardiopulmonary bypass (CBP) the aneurysm was initially resected and then CABG was successfully performed. Although the administration of inotropic agents had already started, the first attempt at weaning off was unsuccessful, CBP was restarted and intra-aortic balloon pumping (IABP) was instituted. In spite of repeatedly attempting and manipulating, this subject never achieved a successful separation from CBP. The fatal outcome was possibly due to multiple embolic events which occurred after induction of anaesthesia and mainly during cannulation.

## 3. Discussion

The first report on this clinical entity was published by Lang and Posselt in 1934 [8]. Since then several cases and studies have been published in the literature, although the atrial septal aneurysm remains a great diagnostic challenge for the clinician. An atrial septal aneurysm (ASA) is a congenital cardiac abnormality that is characterized by a localized bulging of the atrial septum into either or both atria during the cardiac cycle. The majority of ASA can clinically be dormant for a long time but are strongly associated with embolic events and atrial fibrillation. Two-dimensional echocardiography and TEE have made the detection of this abnormality easier and more frequent. TEE is the diagnostic method of choice for ASA. If echocardiography is inconclusive for the diagnosis of an ASA, MRI is the imaging study of choice for further evaluation [9]. According to Olivares-Reyes et al., the ASA can be separated into five types, 1R (the ASA protrudes from the midline of the atrial to the right atrium throughout the cardiorespiratory cycle), 2L (the ASA protrudes from the midline of the atrial septum to the left atrium throughout the cardiorespiratory cycle), 3RL (the maximal excursion of the ASA is toward the right atrium with a lesser excursion toward the left atrium), 4LR (the maximal excursion of the ASA is toward the left atrium with a lesser excursion toward the right atrium), and type 5 (the ASA movement is bidirectional and equidistant to the right as well as to the left atrium during the cardiorespiratory cycle) [1]. In accordance with this classification, our patient belongs to type 1R. 

As mentioned previously, the patient of this case was receiving antiplatelet therapy (aspirin) without any anticoagulation therapy. However, our patient did not experience any thromboembolic event preoperatively. This is in line with Homma et al. who showed that when anticoagulation therapy (warfarin) was compared with aspirin in patients with ASA and PFO, no significant differences were noted in regard with the appearance of thromboembolic events. The difference in our case is the fact that the ASA was a coincidental finding during surgery, and this is the reason why the patient was not receiving any anticoagulants preoperatively. Unfortunately, this ASA was full of thrombi, which were detached as a consequence of surgical manipulations, mainly during cannulation and the patient inevitably experienced a fatal thromboembolic event. Therefore, we think that when we deal with an unexpected finding like ASA we suggest that we perform an alternative method of cannulation and avoid handling the heart.

ASA is usually associated with both atrial and ventricular arrhythmias. The presence of an aneurysm within the interatrial septum may affect the duration of atrial repolarization [3, 6] and is usually associated with the presence of atrial fibrillation (AF) [3, 5]. Indeed, in our case the patient was suffering from AF. Furthermore, data from Morelli et al. suggested that patients with ASA have significantly more runs of ventricular tachycardia than controls [10]. It is unclear, though, if these life threatening arrhythmias are due to the ASA or other structural cardiac abnormalities frequently associated with ASA [11]. 

In our case, a huge ASA could not be detected with the routine preoperative transthoracic echocardiography. This severe abnormality was revealed only when a TEE was performed during surgery. This case reinforces once again the importance of the perioperative TEE for accurate diagnosis of cardiac abnormalities and underlines the need for a detailed cardiac investigation in patients undergoing a cardiac surgery.
